# Serum Iron Status of Under-Five Children with Sickle Cell Anaemia in Lagos, Nigeria

**DOI:** 10.1155/2013/254765

**Published:** 2013-10-31

**Authors:** S. O. Akodu, I. N. Diaku-Akinwumi, O. A. Kehinde, O. F. Njokanma

**Affiliations:** Department of Paediatrics, Lagos State University Teaching Hospital, Ikeja 100001, Nigeria

## Abstract

*Background*. Iron status in patients with sickle cell anaemia is a matter of continuing investigation. *Objective*. This paper aims to determine the serum iron status of under-five, sickle cell anaemia patients. *Methods*. The study spanned from December 2009 to February 2010 at the Consultant Outpatient Clinics involving 97 HbSS subjects and 97 age- and sex-matched HbAA controls. Biochemical iron status was assayed in subjects and controls. *Results*. Age range of the children was seven months to five years, with a mean of 30.6 (±15.97) months. Irrespective of gender, mean serum iron values were higher in HbAA controls than their HbSS counterparts but the observed difference was not significant (*P* = 0.299 and 0.111, resp.). The mean total iron binding capacity values of males and females were also not significantly different for sickle cell anaemia subjects and controls (*P* > 0.05). Males and females with HbAA had significantly lower serum ferritin when compared with their HbSS counterparts. Irrespective of gender, mean transferrin saturation was lower in HbSS subjects but the difference was not statistically significant (*P* > 0.05). *Conclusion*. Children with sickle cell anaemia have higher serum ferritin than controls, implying relatively higher iron content in the reticuloendothelial cells.

## 1. Introduction

Sickle cell anaemia contributes significantly to morbidity and mortality among children in sub-Saharan Africa. Much is known about the disease presentation and end organ manifestation but the iron status in children with Sickle cell anaemia is still a matter of controversy [1].

In children with sickle cell anaemia, chronic haemolysis results in increased availability of iron directly from lysed red cells and also from increased absorption of iron from the gastrointestinal tract [2]. Additionally, the high load of iron provided by multiple blood transfusions [3, 4] would suggest that iron deficiency is unlikely in sickle cell anaemia. However, in some parts of the world, the frequency of blood transfusion among patients is now less as a result of improved management in recent years [5]. Reduced frequency of transfusion implies a reduction in sources of iron and, therefore, increased vulnerability to iron deficiency anaemia. This assertion is buttressed by a study in the USA which suggested that iron deficiency was commoner than expected in untransfused children with sickle cell anaemia [6]. In addition, frequency and need for blood transfusion are not uniform for all children with sickle cell anaemia.

Ferritin is a high-molecular-weight protein that contains approximately 20% iron [7]. It occurs normally in almost all tissues of the body but especially in hepatocytes and reticuloendothelial cells, where it serves as an iron reserve. Ferritin is also present in the serum in minute amounts, where it appears to reflect iron stores in normal individuals. Ferritin plays a significant role in the absorption, storage, and release of iron. As the storage form of iron, ferritin remains in the body tissues until it is needed for erythropoiesis. When needed, the iron molecules are released from the apoferritin shell and bind to transferrin, the circulating plasma protein that transports iron to the erythropoietic cells. Transferrin is the plasma iron transport protein, which binds iron strongly at physiological pH [8]. Transferrin is generally only 25% to 30% saturated with iron [9].

In addition to serum ferritin and transferrin, serum iron and total iron binding capacity constitute indices of iron status in human subjects. The aim of the current study was to study all four biochemical markers in an effort to assess the iron status of children with sickle cell anaemia. It is expected that the findings would add to available information and assist in patient care.

## 2. Materials and Methods

The cross-sectional study was conducted between December 2009 and February 2010 among children with sickle cell anaemia attending the Sickle Cell Disorder Clinic and other Consultant Outpatient Clinics of the Department of Paediatrics, Lagos State University Teaching Hospital, Ikeja in Southwest Nigeria. The Lagos State University Teaching Hospital is an urban tertiary health centre. It is a major referral center serving the whole of Lagos State, which is a major point of entry into Nigeria from different parts of the world and the economic nerve centre of Nigeria.

Approval for the study was obtained from the Ethics Committee of the Lagos State University Teaching Hospital. Consecutive sickle cell anaemia patients aged five years and below, who came for routine follow-up clinic and have satisfied the study criteria, were recruited. Healthy controls were children with genotype “AA”, from the General Outpatient and follow-up clinics, and healthy children attending other specialist clinics (e.g., Paediatric Dermatology Clinic) and were matched for age and sex. Written informed consent was obtained from parents of participants. The study sample size consisted of 194 children, 97 each with haemoglobin genotype SS and AA.

*Inclusion Criteria*

- Aged six months to five years,
- confirmed Hb SS by electrophoresis,
- subjects in steady state, that is, absence of any crisis in the preceding four weeks and absence of any symptom or sign attributable to acute illness [10].

*Exclusion Criteria*

- Denial of consent,
- children on long-term transfusion therapy,
- children who had received a blood transfusion within three months prior to the study,
- children with a history of prematurity or low birth weight.

The inclusion criteria for the controls were the same as for subjects except that the haemoglobin genotype was AA and they had no symptoms or signs attributable to acute illness in the preceding four weeks. Also, the exclusion criteria for the controls were the same as for subjects except that the haemoglobin genotype was AA and they had received iron supplementation within three months prior to recruitment.

Five millilitres of blood were drawn from a convenient peripheral vein into plain tubes. The vacuum tubes were labelled and placed in a cool box containing ice-packs. The samples were protected from light at all times using sheets of black plastic. They were transported to the Research Laboratory of the Department of Paediatrics, Lagos State University College of Medicine.

After centrifugation the serum was separated and stored at minus 20°C until assay. The unsaturated or latent ironbinding capacity (UIBC) was measured by spectrophotometric techniques. Transferrin saturation (expressed as percentage of total iron binding capacity) was calculated using the formula: 100 X the serum iron concentration divided by total iron binding capacity [11]. Serum iron was measured using Iron Ferrozine test kit (Biosystems, Spain) while the TIBC was measured by using Iron/Total Iron Binding Capacity reagent set (TECO Diagnostics, USA). Serum ferritin was measured by using human ferritin enzyme immunoassay test kit (Diagnostic Automation, USA).

Social class was determined from occupation and educational attainment of parents using the scheme proposed by Oyedeji [12]. The subjects were classified into one of five classes (I–V) in descending order of social privilege. Study subjects were then further stratified into upper (classes I and II), middle (class II), and lower (classes IV and V) socioeconomic groups. The data was analyzed using the Statistical Package for Social Science (SPSS) version 17.0. Level of significance was set at *P* < 0.05.

## 3. Results

A total of 194 children, 97 each with genotype SS and AA, respectively, were recruited. The demographic characteristics of the study subjects are given in Table 1. Overall, the age of the subjects ranged from seven months to sixty months with a mean of 30.61 (±15.97) months: 32.05 ± 16.12 months and 29.18 ± 15.77 months for SS subjects and AA controls, respectively (Mann-Whitney *U* = 4143.50, *P* = 0.151). The median age was 25.00 months and 26.00 months in SS subjects and AA controls, respectively. Ninety-six (49.5%) of the study subjects belonged to the upper socioeconomic strata (socioeconomic indices I and II), while 34.5% and 16.0% belong to the middle (socioeconomic index III) and lower (socioeconomic index IV and V) socioeconomic strata, respectively.

### 3.1. Serum Iron, Serum Ferritin, Total Iron Binding Capacity, and Transferrin Saturation Profile of Study Subjects

Comparisons of serum iron, serum ferritin, total iron binding capacity (TIBC), and transferrin saturation between SS subjects and AA controls are shown in Table 2. In both males and females, the mean serum iron values were higher among haemoglobin genotype AA controls than their SS counterparts but the observed difference was not significant (*P* = 0.872 and 0.166 for males and females, resp.). The mean TIBC values of males and females were also not significantly different for Hb AA subjects and their counterparts with Hb SS (*P* > 0.05).

Males and females with haemoglobin genotype AA had significantly lower serum ferritin when compared with their haemoglobin genotype SS counterparts (*P* = 0.000 for each gender). Irrespective of gender, the mean serum iron, mean serum ferritin TIBC and transferrin saturation was lower in subjects without history of blood transfusion prior to commencement of study but the difference was not statistically significant (*P* > 0.05).

Sixty-four of study subjects with haemoglobin genotype SS have never had blood transfusion prior to commencement of the study. Ten of the thirty-three sickle cell anaemia subjects who have a history of previous blood transfusions reported two or more transfusion episodes (nine had two episodes of previous blood transfusions while one reported blood transfusions on three different occasions). Comparisons of serum iron, serum ferritin, total iron binding capacity (TIBC), and transferrin saturation between sickle cell anaemia patients with past history of blood transfusion and those who have never been transfused are shown in Table 3. Irrespective of gender, the mean serum iron, mean serum ferritin, TIBC, and transferrin saturation were lower in subjects with history of blood transfusion prior to commencement of study but the difference was not statistically significant (*P* > 0.05).

### 3.2. Comparison of Serum Ferritin and Transferrin Saturation between Sickle Cell Anaemia Patients with Past History of Blood Transfusion and Those Who Have Never Been Transfused

Table 4 shows the comparison of criteria for diagnosing iron deficiency among children with sickle cell anaemia with previous history of blood transfusion prior to commencement of study. The diagnosis of iron deficiency was established based on the following criteria: transferrin saturation (Ts) < 16% [13, 14] or serum ferritin (SF) < 25 ng/dL [13, 14]. All the sickle cell anaemia subjects with serum ferritin <25 ng/dL reported no past history of blood transfusion while two-thirds of the sickle cell anaemia subjects with serum ferritin ≥25 ng/dL reported no past history of blood transfusion. Similarly, almost two-thirds and three-quarters of subjects with haemoglobin genotype SS with transferrin saturation ≥16% and <16%, respectively, had no blood transfusion prior to commencement of the study. However, these observed differences were not significant (*P* = 0.864, *P* = 0.085 for serum ferritin and transferrin saturation, resp.).

### 3.3. Haemoglobin Concentration Distribution of Study Subjects

The comparison of the mean haemoglobin concentration values between HbSS subjects and HbAA controls is shown in Table 5. Overall, the mean haemoglobin concentration was significantly higher among HbAA controls than their counterparts with sickle cell anaemia. This pattern was observed at all age groups except subjects >2 years to 3 years.

## 4. Discussion

Subjects with haemoglobin genotype SS had a somewhat lower mean serum iron concentration than haemoglobin genotype AA controls. This finding is consistent with that of Jeyakumar et al. [13] in Ibadan, Nigeria. Serum iron concentration is a balance between intake on the one hand and excretion as well as increased utilization on the other. Since there is no empirical reason to believe that HbSS subjects had lower dietary intake of iron, attention will have to be focused on excretion. Thus it is attractive to agree with Jeyakumar et al. [13] that sickle cell anaemia patients lose excessive amounts of iron. The explanation may be found in the study by Koduri [14] in which it was stated that one-third of the haemolysis in sickle cell anaemia subjects takes place in the intravascular space and is associated with excessive urinary loss of iron. As far as the current study is concerned, however, these explanations remain conjectural because urinary loss of iron was not investigated.

The mean serum ferritin was significantly higher among SS subjects than AA controls. Serum ferritin is known to reflect mainly reticuloendothelial iron stores [5]. It is considered to be a sensitive indicator of body iron stores and thus elevated serum ferritin concentrations may reflect possible high body iron stores. A higher value of serum ferritin in children with sickle cell anaemia may be due to presence of increased iron in the reticuloendothelial cells resulting from the excessive breakdown of haemoglobin.

In absolute terms, the mean serum ferritin value of less than 220 *μ*g/L in our HbSS subjects was lower than 367 *μ*g/L reported by Hussain et al. [5] in London. The observed difference is possibly an effect of age range of study subjects. It has been shown that serum ferritin levels may be modified by age [15]. Even within the relatively narrow age range involved in the current study, some positive correlation was demonstrated between age and serum ferritin levels. Thus the study by Hussain et al. [5] which included much older children (up to 15 years) would expectedly report higher ferritin levels. Serum ferritin may also be affected by genetic factors [16]. The current study recruited black children with sickle cell anaemia while Hussain et al. [5] recruited white children with conceivably different genetic composition. In addition, factors including overall nutrition could potentially affect ferritin levels.

In the present study, high serum ferritin level was accompanied by reduced serum iron in sickle cell anaemia subjects. The reason for this pattern of results was beyond the scope of the present study. It is however known that high iron stores as implied from high serum ferritin are associated with increased release of hepcidin which in turn leads to reduced serum iron [17]. Hepcidin is a 25 amino acid peptide made by hepatocytes [18]. Its production is enhanced by high iron stores and inflammation [17, 18]. It is, therefore, also attractive to speculate that sickle cell anaemia, being a state of chronic inflammation [19], would be associated with elevated hepcidin levels with consequent lower serum iron.

The TIBC measures the availability of iron binding sites [20]. Extracellular iron is transported in the body bound to transferrin, which is the primary iron-transport protein [20]. Hence, TIBC indirectly measures transferrin levels, which increase as stored iron decreases [20]. The present study showed that the mean TIBC was higher among AA controls than in SS subjects. The possible explanation may be the presence of high body iron stores among subjects with sickle cell anaemia revealed by higher serum ferritin values.

Similarly, we observed higher mean transferrin saturation among AA controls than in SS subjects irrespective of gender. Further, both haemoglobin SS subjects and haemoglobin AA controls had mean values within the normal range of 25% to 30% [21] but with haemoglobin SS in the lower band. The finding is consistent with the observation of slightly lower serum concentration of iron in sickle cell anaemia subjects in the current study. The explanation for the higher mean transferrin saturation among HbAA controls is not farfetched. The transferrin saturation is a measure of amount of iron bound to the protein transferrin and reflects iron transport. As the body iron stores are depleted, the transferrin saturation rises. It has been revealed in the current study that children with sickle cell anaemia have higher body iron stores potentially from increased red cell turnover and from blood transfusions.

Our centre does not have a chronic transfusion programme for sickle cell anaemia patients. Rather, blood transfusion is offered as needed when anaemia is severe. In consequence, the number of patients who had ever received transfusions was relatively small. Even then, very few of them had received enough units of blood transfusion to potentially affect their iron status. The ability of the study to test the influence of blood transfusion was therefore highly restricted.

Overall, the mean serum ferritin was significantly higher among children with sickle cell anaemia compared to haemoglobin AA controls which may be due to presence of increased iron in the reticuloendothelial cells because of the excessive breakdown of haemoglobin. Age had poor linear relationship with the transferrin saturation and serum ferritin. Further studies are needed to further explore the additional factors such as body composition that may influence iron status in children with sickle cell anaemia.

## Figures and Tables

**Table 1:** Demographic characteristics of the study population.

Characteristics	AA	SS	ALL
Gender			
Male	49 (50.5)	49 (50.5)	98 (50.5)
Female	48 (49.5)	48 (49.5)	96 (49.5)
Age group (years)			
0-1	15 (15.5)	6 (6.2)	21 (10.8)
>1-2	33 (34.0)	42 (43.3)	75 (38.7)
>2-3	17 (17.5)	11 (11.3)	28 (14.4)
>3-4	18 (18.6)	15 (15.5)	33 (17.0)
>4-5	14 (14.4)	23 (23.7)	37 (19.1)
Socioeconomic index			
I	18 (18.6)	13 (13.4)	31 (16.0)
II	36 (37.1)	29 (29.9)	65 (33.5)
III	27 (27.8)	40 (41.2)	67 (34.5)
IV	15 (15.5)	14 (14.9)	29 (14.9)
V	1 (1.0)	1 (1.1)	2 (1.0)

**Table 2:** Serum iron, serum ferritin, total iron binding capacity, and transferrin saturation profile of study subjects.

	SS Mean (SD)	AA Mean (SD)	M-W *U*-value	*P* value
Serum iron (*µ*g/dL)				
Males	67.4 (34.54)	78.3 (64.28)	960.50	0.872
Female	67.3 (47.44)	85.8 (63.36)	569.50	0.166
Male and female	67.4 (40.78)	81.6 (63.61)	2999.50	0.264
Serum ferritin (ng/dL)				
Male	170.4 (107.53)	62.0 (61.91)	353.00	0.000*
Female	211.5 (97.48)	47.4 (42.30)	87.00	0.000*
Male and female	189.9 (104.28)	55.5 (54.20)	823.50	0.000*
Total iron binding capacity (*µ*g/dL)				
Male	253.2 (86.73)	274.6	851.50	0.496
Female	265.4 (81.99)	293.5 (95.70)	568.50	0.226
Male and female	259.0 (84.16)	283.1	2817.00	0.18
Transferrin saturation (%)				
Male	27.0 (12.14)	29.1 (17.37)	918.00	0.911
Female	24.9 (13.40)	28.5 (16.55)	614.00	0.474
Male and female	26.0 (12.70)	28.8 (16.91)	3089.50	0.697

M-W: Mann Whitney.

*Statistically significant.

**Table 3:** Serum iron, serum ferritin, total iron binding capacity, and transferrin saturation profile of sickle cell anaemia subjects according to history of blood transfusion.

Variable	Past history of blood transfusion		M-W *U*-Value	*P* value
	Yes Mean (SD)	No Mean (SD)		
Serum iron (*µ*g/dL)	77.27 (48.28)	62.08 (35.62)	509.50	0.156
Serum ferritin (ng/dL)	202.85 (100.94)	183.14 (106.35)	574.00	0.045
Total iron binding capacity (*µ*g/dL)	279.50 (97.00)	248.69 (75.98)	432.00	0.086
Transferrin saturation (%)	27.77 (13.38)	25.11 (12.40)	498.00	0.351

M-W: Mann Whitney.

**Table 4:** Serum ferritin and transferrin in subjects with sickle cell anaemia according to past history of blood transfusion.

Variable	Past history of blood transfusion		Total	*P* value
	Yes	No		
Serum iron (*µ*g/dL)				0.519^+^
<25	0 (0.0)	3 (100.0)	3 (100.0)	
≥25	33 (35.1)	61 (64.9)	94 (100.0)	
Total	**33 (34.0)**	**64 (66.0)**	**97**	

Transferrin saturation (%)				0.613
<16	6 (28.6)	15 (71.4)	21 (100.0)	
≥16	27 (35.6)	49 (64.4)	76 (100.0)	
Total	**33 (34.0)**	**64 (66.0)**	**97**	

Values in parenthesis are % of column total.

^+^Chi-square test (*χ* ^2^).

**Table 5:** Haemoglobin concentration distribution of study subjects.

Age group (years)	AA Mean (SD)	SS Mean (SD)	*t*-value	*P* value
0-1	8.79 (1.24)	6.62 (1.65)	3.125	0.006*
>1-2	9.73 (2.21)	6.60 (1.05)	7.863	0.000*
>2-3	9.38 (0.80)	7.72 (3.30)	2.014	0.054
>3-4	9.84 (1.05)	6.55 (1.56)	7.217	0.000*
>4-5	10.51 (0.90)	6.85 (1.34)	9.006	0.000*
ALL	9.66 (1.58)	6.79 (1.64)	12.279	0.000*

*Statistically significant.
